# Salicylic acid signal transduction: the initiation of biosynthesis, perception and transcriptional reprogramming

**DOI:** 10.3389/fpls.2014.00697

**Published:** 2014-12-09

**Authors:** Carolin Seyfferth, Kenichi Tsuda

**Affiliations:** Department of Plant Microbe Interactions, Max Planck Institute for Plant Breeding Research, Cologne, Germany

**Keywords:** calcium, ICS1, NPR1, plant immunity, salicylic acid, SA perception, transcriptional reprogramming

## Abstract

The phytohormone salicylic acid (SA) is a small phenolic compound that regulates diverse physiological processes, in particular plant resistance against pathogens. Understanding SA-mediated signaling has been a major focus of plant research. Pathogen-induced SA is mainly synthesized via the isochorismate pathway in chloroplasts, with ICS1 (ISOCHORISMATE SYNTHASE 1) being a critical enzyme. Calcium signaling regulates activities of a subset of transcription factors thereby activating nuclear *ICS1* expression. The produced SA triggers extensive transcriptional reprogramming in which NPR1 (NON-EXPRESSOR of PATHOGENESIS-RELATED GENES 1) functions as the central coactivator of TGA transcription factors. Recently, two alternative but not exclusive models for SA perception mechanisms were proposed. The first model is that NPR1 homologs, NPR3 and NPR4, perceive SA thereby regulating NPR1 protein accumulation. The second model describes that NPR1 itself perceives SA, triggering an NPR1 conformational change thereby activating SA-mediated transcription. Besides the direct SA binding, NPR1 is also regulated by SA-mediated redox changes and phosphorylation. Emerging evidence show that pathogen virulence effectors target SA signaling, further strengthening the importance of SA-mediated immunity.

## INTRODUCTION

The phytohormone salicylic acid (SA) is a small phenolic compound that functions as an important signaling molecule during plant immunity (Vlot et al., 2009; Robert-Seilaniantz et al., 2011; Pieterse et al., 2012). Since constitutive SA accumulation is often associated with stunted plant growth, resulting in reduction of plant fitness (Ishihara et al., 2008; Pajerowska-Mukhtar et al., 2012; Chandran et al., 2014), SA biosynthesis and SA-mediated signaling are tightly controlled.

The plant immune system comprises multiple layers, such as pattern-triggered immunity (PTI) and effector-triggered immunity (ETI; Jones and Dangl, 2006; Tsuda and Katagiri, 2010). PTI is triggered by recognition of common microbial components (MAMPs, microbe-associated molecular patterns), such as bacterial flagellin or the fungal cell wall component chitin (Boller and Felix, 2009; Macho and Zipfel, 2014). MAMP recognition stimulates generation of reactive oxygen species, intracellular calcium influx, transient activation of mitogen-activated protein kinases (MAPKs), and the production of SA (Tsuda et al., 2008a,b; Tsuda and Katagiri, 2010). Virulent pathogens, for example, the bacterial pathogen *Pseudomonas syringae* pv. *tomato* DC3000 (*Pto* DC3000), however, can suppress PTI in *Arabidopsis* and tomato by effectors, injected via bacterial secretion systems into the plant cell (Lohou et al., 2013; Xin and He, 2013). Recent studies identified various effectors that interfere with SA signaling (Uppalapati et al., 2007; Djamei et al., 2011; Caillaud et al., 2013; Jiang et al., 2013; Rabe et al., 2013; Gimenez-Ibanez et al., 2014; Liu et al., 2014), highlighting the importance of SA signaling for plant immunity. To regain resistance, plants have acquired intracellular receptors [resistance (R) proteins], which induce the second layer of defense after effector recognition, termed ETI (Eitas and Dangl, 2010; Bonardi and Dangl, 2012; Jacob et al., 2013). Activation of ETI also induces SA accumulation and MAPK activation, which are also important for resistance against pathogens during ETI (Tsuda et al., 2013). Additionally, SA has vital roles in establishing systemic acquired resistance (SAR), a form of long-term and broad-spectrum resistance throughout the entire plant after local pathogen infection (Wang et al., 2006; Fu and Dong, 2013).

In this review, we summarize SA signal transduction from regulation of biosynthesis, perception, to transcriptional reprogramming during plant immunity. We also discuss compensation mechanisms that would provide robust immunity once SA signaling is compromised, for example, by pathogen effector attack. SA signaling pathway is highly interconnected with other phytohormone signaling such as mediated by jasmonates (JA), ethylene, and abscisic acid (Robert-Seilaniantz et al., 2011; Pieterse et al., 2012; Derksen et al., 2013). For example, JA and ethylene signaling negatively regulate SA biosynthesis at the transcriptional level (Chen et al., 2009; Zheng et al., 2012). However, discussions on these are beyond the scope of this review.

## THE BIOSYNTHESIS OF SA IN PLANTS

### BIOSYNTHETIC PATHWAYS

Two major SA biosynthetic pathways in plants were identified: the isochorismate (IC) and the phenylalanine ammonia-lyase (PAL) pathways. Both pathways commonly utilize chorismate, the end product of the shikimate pathway, to produce SA (Dempsey et al., 2011). IC synthase (ICS) and PAL are critical enzymes for these pathways, respectively. Homologs of *ICS* and *PAL* genes are present throughout the plant kingdom, including *Arabidopsis*, tobacco, tomato, populus, sunflower, and pepper (Wildermuth et al., 2001; Cochrane et al., 2004; Uppalapati et al., 2007; Catinot et al., 2008; Yuan et al., 2009; Sadeghi et al., 2013; Kim and Hwang, 2014), suggesting the importance of these SA biosynthesis pathways to survive during the course of evolution. In *Arabidopsis*, mutations in *ICS1* lead to an almost complete loss of pathogen-induced SA accumulation (Wildermuth et al., 2001). However, *Arabidopsis* quadruple *PAL* mutants, in which PAL activity is reduced to 10%, also show lower SA accumulation (50%) compared to the wild type upon pathogen infection (Huang et al., 2010). Thus, while contribution of the PAL pathway is evident, the IC pathway is the major route for SA biosynthesis during plant immunity.

In chloroplasts, ICS catalyzes the conversion of chorismate into IC (Wildermuth et al., 2001; Strawn et al., 2007; Garcion et al., 2008), which is further converted to SA (Dempsey et al., 2011). In some bacteria, conversion of IC to SA is catalyzed by IC pyruvate lyases (IPLs; Dempsey et al., 2011). However, plant genomes encode no homologous genes to bacterial *IPLs*. Expression of bacterial enzymes catalyzing this conversion together with ICS in chloroplasts leads to constitutive accumulation of SA (Verberne et al., 2000; Mauch et al., 2001). Thus, it is conceivable that plants have yet-determined gene(s) whose product(s) possess IPL activity in chloroplasts. However, metabolic enzymes such as the acyl acid amido synthetase GH3.12 [also known as PBS3/WIN3/GDG1 (AVRPPHB SUSCEPTIBLE 3/HOPW1-INTERACTING 3/GH3-LIKE DEFENSE GENE 1); Nobuta et al., 2007; Zhang et al., 2007; Okrent et al., 2009; Westfall et al., 2010, 2012] and the acyltransferase EPS1 (ENHANCED PSEUDOMONAS SUSCEPTIBILITY 1; Zheng et al., 2009) are involved in SA accumulation, perhaps by providing SA precursors or regulatory molecules for SA biosynthesis. Thus, SA biosynthesis may be more complex in plants compared to bacteria. SA export from chloroplasts is mediated by the MATE-transporter EDS5 (ENHANCED DISEASE SUSCEPTIBILITY 5; Serrano et al., 2013). This export seems important for SA accumulation and distribution in the cell since SA accumulation is compromised in *eds5* mutants (Nawrath et al., 2002; Ishihara et al., 2008).

### REGULATION OF SA BIOSYNTHESIS

**FIGURE 1:**
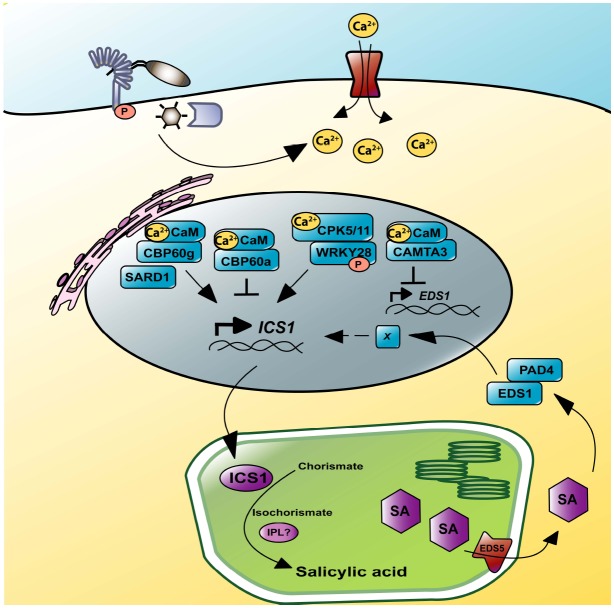
**Regulation of SA accumulation by calcium signaling.** MAMP or effector recognition increases intracellular Ca^2+^ concentrations thereby regulating calcium sensor proteins, such as CaM and CDPKs. The CaM-binding transcription factors CBP60g and CBP60a are positive and negative regulators of *ICS1* transcription, respectively. A homolog of CBP60a/g, SARD1, is not a CaM-binding protein but functions redundantly with CBP60g for *ICS1* transcription. WRKY28, whose DNA-binding activity is regulated by the CDPKs CPK5 and CPK11, also contributes to *ICS1* expression. ICS1 mediates SA production in chloroplasts, by conversion of chorismate into the SA-precursor isochorismate. SA may be transported through the MATE-transporter EDS5 into the cytosol. The EDS1/PAD4 complex contributes to the positive feedback loop of SA accumulation. Repression of *EDS1* transcription by the Ca^2+^/CaM-binding transcription factor CAMTA3 represents a fine-tuning mechanism for SA accumulation.

Salicylic acid biosynthesis is tightly regulated since constitutive SA accumulation has negative impacts on plant fitness (Ishihara et al., 2008; Pajerowska-Mukhtar et al., 2012; Chandran et al., 2014). Accumulating evidence show that transcriptional control of *ICS1* by calcium signaling is key for the initiation of SA biosynthesis (Figure 1). The concentration of calcium ions (Ca^2+^) in the cytosol transiently increases upon immune receptor activation through Ca^2+^ channels. Elevation of intracellular Ca^2+^, called Ca^2+^ signature, is decoded by Ca^2+^ sensor proteins, such as calmodulin (CaM) and Ca^2+^-dependent protein kinases (CDPKs; Dodd et al., 2010; Boudsocq and Sheen, 2013; Poovaiah et al., 2013; Schulz et al., 2013). Binding of CaM regulates target protein activities thereby relaying Ca^2+^ signatures to downstream responses. During *Arabidopsis* immunity, the CaM-binding transcription factor CBP60g (CALMODULIN BINDING PROTEIN 60g) and its homolog SARD1 (SYSTEMIC ACQUIRED RESISTANCE DEFICIENT 1) control *ICS1* transcription (Wang et al., 2009, 2011; Zhang et al., 2010; Wan et al., 2012). CaM-binding is required for CBP60g function, whereas SARD1 does not appear to be a CaM-binding protein (Wang et al., 2009). Despite this difference, CBP60g and SARD1 are partially redundant for *ICS1* expression and SA accumulation during immunity. However, dual regulation of *ICS1* transcription by CBP60g and SARD1 seems important for temporal dynamics of SA biosynthesis: CBP60g mainly contributes to SA biosynthesis at early stages after *P. syringae* infection while SARD1 does at late stages (Wang et al., 2011). Another close homolog of CBP60g, CBP60a, negatively regulates *ICS1* expression upon CaM-binding (Truman et al., 2013). Conceivably, upon pathogen attack, CBP60g and SARD1 bind to the *ICS1* promoter and activate its expression, at least partly by removing the negative regulator CBP60a from the *ICS1* promoter.

Unlike CaM, CDPKs have both intrinsic Ca^2+^ sensing and responding sites thereby allowing individual CDPK proteins to relay Ca^2+^ signatures to downstream components via phosphorylation events. Recently, the CDPKs, CPK4, 5, 6, and 11, were shown to re-localize to the nucleus, and to interact with and phosphorylate the WRKY transcription factors, WRKY8, 28, and 48, during ETI mediated by the plasma membrane-associated immune receptors RPS2 (RESISTANCE TO P.SYRINGAE 2) or RPM1 (RESISTANCE TO P.SYRINGAE PV MACULICULA 1; Gao et al., 2013). Mutants in *WRKY8* or *WRKY48* are compromised in pathogen-induced *ICS1* expression. Furthermore, WRKY28 directly interacts with the *ICS1* promoter (van Verk et al., 2011), which might be regulated through phosphorylation by CPK4, 5, 6, or 11. Collectively, these results suggest that during ETI, these CDPKs relay Ca^2+^ signatures to activate *ICS1* transcription via WRKY transcription factors.

Besides *ICS1* regulation, calcium signaling also affects the maintenance of SA accumulation through transcriptional regulation of *EDS1 (ENHANCED DISEASE SUSCEPTIBILITY 1*; Du et al., 2009), encoding a central regulator of the positive feedback loop of SA accumulation (Feys et al., 2001). A CaM-binding transcription factor, CAMTA3/SR1 (CALMODULIN BINDING TRANSCRIPTION ACTIVATOR 3/SIGNAL-RESPONSIVE GENE 1), binds to the *EDS1* promoter to repress its transcription, and mutants of *CAMTA3/SR1* show elevated SA levels and enhanced immunity against *P. syringae* and the fungal pathogen *Botrytis cinerea*. Combinatorial mutant analysis indicates that CAMTA3/SR1 and its homologs CAMTA1/2 also suppress expression of *CBP60g*, *SARD1*, and *ICS1* (Kim et al., 2013). Thus, the three CAMTA homologs coordinately suppress SA accumulation, but it remains unknown if the CAMTA transcription factors directly target the promoters of *CBP60g*, *SARD1*, and *ICS1*. It was recently shown that a CAMTA3/SR1-interacting protein links CAMTA3/SR1 to ubiquitin-mediated protein degradation thereby enhancing *EDS1* expression and immunity against *P. syringae* (Zhang et al., 2014).

In summary, these results clearly indicate the importance of Ca^2+^ signaling in regulation of SA accumulation during immunity through transcriptional regulation of genes involved in SA biosynthesis and maintenance. However, how plants spatiotemporally coordinate positive and negative regulators of SA biosynthesis and accumulation remains to be investigated.

## SA PERCEPTION

Identification of SA receptor(s) has been one of the major research interests for the last two decades. Considering its diverse functions in environmental stress response, plants may have multiple SA receptors. Indeed, biochemical approaches identified a number of SA-interacting proteins, and activities of these proteins were affected by SA-binding (Chen and Klessig, 1991; Chen et al., 1993; Durner and Klessig, 1995; Du and Klessig, 1997; Slaymaker et al., 2002; Kumar and Klessig, 2003; Forouhar et al., 2005; Park et al., 2009; Tripathi et al., 2010; Tian et al., 2012; Moreau et al., 2013). However, these SA-binding proteins do not fully explain SA response including SA-mediated transcriptional reprogramming. Recently, the three NPR (NON-EXPRESSOR of PATHOGENESIS-RELATED GENES) family members, NPR1, NPR3, and NPR4, were identified as *bona fide* SA receptors in *Arabidopsis* (Fu et al., 2012; Wu et al., 2012). In this section, we discuss how these NPR proteins function as SA receptors.

**FIGURE 2:**
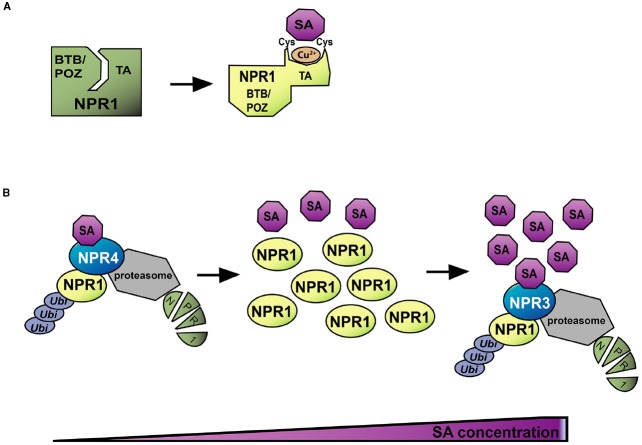
**Models for SA perception. (A)** Direct SA binding to NPR1 modulates its activity. In unstressed conditions, the C-terminal transactivation domain of NPR1 is repressed by the N-terminal BTB/POZ domain, keeping NPR1 in an inactive state (green). NPR1 perceives SA through Cys^521/529^ via the transition metal copper, which triggers a conformation change of NPR1, resulting in de-repression of the transactivation domain and activation of NPR1 (yellow). **(B)** NPR1 accumulation is regulated by SA through the SA receptors NPR3 and NPR4. Pathogen infection triggers SA accumulation. In the case of low SA, the SA-receptor NPR4 triggers NPR1 degradation through the 26S proteasome. When SA levels are intermediate, NPR1 protein accumulates. High SA-concentrations trigger the SA receptor NPR3-mediated NPR1 degradation. Thus, only intermediate levels of SA achieve NPR1 accumulation thereby activating SA-mediated transcriptional reprogramming.

NPR1 is a master regulator of SA-mediated transcriptional reprogramming and immunity, functioning as a transcriptional coactivator (Pajerowska-Mukhtar et al., 2013). NPR1 comprises a BTB/POZ (broad-complex, tramtrack, and bric-à-brac/poxvirus and zinc-finger) domain, an ankyrin repeat domain, and a nuclear localization sequence. Mutations in *NPR1* lead to an almost complete loss of SA-mediated transcriptional reprogramming and great susceptibility to (hemi)-biotrophic pathogens (Shah et al., 1997; Volko et al., 1998; Dong, 2004). Therefore, it was not surprising but sensational that Wu et al. (2012) found NPR1 to be a *bona fide* SA receptor (Figure 2A). Using an equilibrium method, they showed that *Arabidopsis* NPR1 directly binds SA (Kd = 140 nM), but not inactive structural analogs, through Cys^521/529^ via the transition metal copper. Consistently, Cys^521/529^ were previously identified as key amino acid residues for *Arabidopsis* NPR1 function (Rochon et al., 2006). Biochemical approaches indicate that SA-binding triggers a conformational change in NPR1. Further protein deletion analyses suggest that the C-terminal transactivation domain of NPR1 is intramolecularly inhibited by the N-terminal BTB/POZ domain and that SA-binding releases the transactivation domain from BTB/POZ suppression. Thus, the study established a model with NPR1 as an SA receptor that also functions as a master signal transducer of SA signaling. However, Cys^521/529^ are not conserved among plant species, raising an issue of the evolutionary significance of the SA perception mechanism via NPR1. In addition, another study showed that NPR1 does not bind SA in a conventional non-equilibrium ^3^H-SA binding assay (Yan and Dong, 2014). Instead, Fu et al. (2012) identified two homologs of NPR1, NPR3 and NPR4, as SA receptors (Figure 2B; Fu et al., 2012). NPR1 is subject to degradation via the 26S proteasome pathway in the absence of SA (Spoel et al., 2009). Once SA increases upon pathogen infection, NPR1 is stabilized. However, full induction of SA-responsive genes also requires NPR1 turnover. Thus, regulation of NPR1 protein level is critical for SA response. Fu et al. (2012) found that NPR3 and NPR4 interact with NPR1 and are required for NPR1 degradation (Fu et al., 2012). NPR4 has a high SA affinity (Kd = 46 nM) whereas NPR3 shows a low affinity (Kd = 981 nM), suggesting differential regulations of NPR1 by NPR3 and NPR4. Interestingly, SA disrupts NPR1–NPR4 interaction, but facilitates NPR1-NPR3 interaction. These observations support a model in which NPR3 and NPR4 create an NPR1 protein concentration gradient in order to regulate NPR1–mediated transcription: in the absence of SA, NPR4-mediated NPR1 degradation prevents NPR1 accumulation whereas high SA levels also prevent NPR1 accumulation due to NPR3. Thus, NPR1-mediated signaling is active only at intermediate SA levels. This model is consistent with the observation that NPR1 protein highly accumulates at sites surrounding the infection site in a leaf. These regions are supposed to contain intermediate SA levels, while the infection site may have too high SA levels. Although this model is attractive, further validation is required.

Collectively, two alternative but not exclusive SA perception mechanisms in plant cells were identified, but further research is still required to address fundamental questions. For example, the subcellular location(s) of SA perception have not been addressed yet. The nuclear NPR1 pool is necessary for SA-mediated transcription (Mou et al., 2003). Consistently, NPR3 and NPR4 are nuclear proteins, and therefore SA is likely perceived by them in the nucleus to regulate nuclear NPR1 amount. On the other hand, the cytosolic NPR1 pool may regulate cross-talk between SA- and JA-mediated transcriptional reprogramming (Spoel et al., 2003), suggesting that SA is also perceived in the cytosol. Since SA perception by nuclear NPR3 and NPR4 does not explain this observation, cytosolic NPR1 activity may be regulated by the direct SA binding.

## SA-MEDIATED TRANSCRIPTIONAL REPROGRAMMING

NPR1 controls expression of more than 95% of the responsive genes to the SA-analog benzothiadiazole (BTH; Wang et al., 2006). Functional regulation of NPR1 is not only mediated by the direct SA binding, but also by SA-triggered redox changes (Mou et al., 2003). In the absence of SA, NPR1 is present as an oligomer formed through intermolecular disulfide bonds. SA triggers changes in the cellular redox potential, thereby reducing cysteine residues in NPR1 through the thioredoxins TRXh3 and TRXh5, resulting in monomerization of NPR1 (Tada et al., 2008). Mutations in the cysteine residues (Cys^82^ or Cys^216^) lead to constitutive monomerization and nuclear accumulation of NPR1, resulting in activation of *PR1* expression (Mou et al., 2003). Nuclear accumulation of NPR1 triggered by SA can be explained by stabilization of nuclear NPR1 or translocation of the NPR1 monomer from the cytosol to the nucleus. Thus, SA-triggered NPR1 monomerization and nuclear accumulation are important steps for NPR1-mediated transcription. However, forced nuclear localization of NPR1 is not sufficient for transcriptional reprogramming, as the presence of SA is additionally required for full *PR1* induction (Kinkema et al., 2000; Spoel et al., 2003). This can be explained by the observation that SA-binding triggers the NPR1 conformational change thereby allowing NPR1 to regulate gene expression (Wu et al., 2012). Additional regulation of NPR1 involves phosphorylation (Spoel et al., 2009). SA triggers phosphorylation of NPR1 at the N-terminus (Ser^11/15^) in the nucleus via yet-determined kinase(s). NPR1 phosphorylation contributes to its recruitment to a ubiquitin ligase, resulting in proteasome-mediated NPR1 degradation. This degradation is required for the proper transcriptional control by NPR1, perhaps by allowing fresh NPR1 to reinitiate the next cycle of transcription.

**FIGURE 3:**
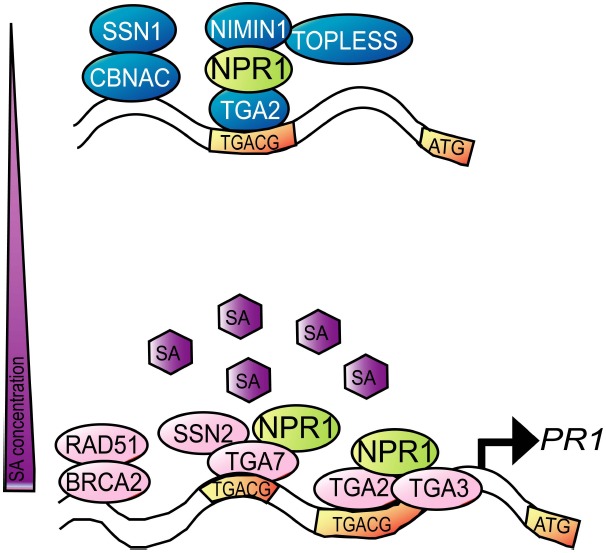
**SA-mediated transcriptional regulation of PR1 through NPR1.** In the absence of SA, repression of *PR1* expression can be achieved by repressor complexes (blue), such as the TGA2-NPR1-NIMIN complex through the co-repressor TOPLESS or the CBNAC-SNI1 complex. SA triggers a conformational change of NPR1 and dissociation of NIMIN1, resulting in forming activator complexes (pink) including TGA transcription factors and SSN2. The DNA repair proteins BRCA2 and RAD51 are also involved in SA-mediated transcription.

NPR1 regulates transcription of SA-responsive genes through interactions with specific transcription factors (Figure 3). Identified major transcription factors belong to a subclass of the basic leucine zipper transcription factor family, TGA (Gatz, 2013). The *Arabidopsis* genome encodes 10 TGA transcription factors, which are structurally divided into five subgroups and all bind the consensus DNA sequence TGACG. Yeast-two-hybrid analyses with NPR1 and TGA transcription factors show interaction specificity for clade II TGAs (TGA2/TGA5/TGA6) and TGA3 (clade III; Zhou et al., 2000; Hepworth et al., 2005). Genetic analysis reveal that TGA2, TGA5, and TGA6 repress *PR1* transcription in the absence of SA, but on the other hand are required for *PR1* induction in the presence of SA (Zhang et al., 2003). In the absence of SA, TGA2 binds to the *PR1* promoter thereby repressing its transcription (Rochon et al., 2006; Boyle et al., 2009). An NPR1-interacting protein, NIMIN1 (NPR1/NIM1-INTERACTING PROTEIN 1), can form a ternary complex with TGA2 through NPR1 at least in yeast (Weigel et al., 2005). Transcriptional repression by TGA2 may be achieved through NIMIN1 interacting with a transcriptional co-repressor, TOPLESS (Braun et al., 2011). Conceivably, SA allows NPR1 to form a different complex with TGA2 and other TGA factors, such as TGA3 thereby activating *PR1* transcription (Johnson et al., 2003). The NIMIN1–NPR1–TGA2 complex is dissociated in the presence of SA in yeast (Hermann et al., 2013). Thus, NIMIN1 dissociation from the NPR1–TGA transcriptional complex by SA may contribute to activation of the NPR1–TGA transcriptional complex. This transcriptional activation may be relayed through specific mediator subunits, such as the Mediator subunit MED15, since *med15* mutants are insensitive to SA (Canet et al., 2012).

A suppressor screen of *npr1* identified SNI1 (SUPPRESSOR OF *NPR1* INDUCIBLE 1) as another repressor of SA-responsive genes (e.g., *PR1*) in unstressed conditions (Li et al., 1999; Mosher et al., 2006). SNI1-mediated transcriptional repression may be achieved through the CaM-binding NAC (NAM, ATAF1,2, CUC2) transcription repressor CBNAC, since SNI1 directly interacts with CBNAC and enhances CBNAC-binding activity to the *PR1* promoter (Kim et al., 2012). Upon SA treatment, SNI1 is dissociated from the *PR1* promoter and replaced by the DNA repair protein SSN2 (Song et al., 2011). Although SSN2 contains a DNA-binding domain, its binding to the *PR1* promoter requires NPR1 and the transcription factor TGA7. These results suggest that SA triggers NPR1 activation through nuclear accumulation and conformational change, resulting in the formation of a TGA7–NPR1–SSN2 complex that activates *PR1* transcription. Additional DNA repair proteins, such as BRCA2A (BREAST CANCER 2A) and RAD51D, are also functionally associated with SA-mediated transcription (Durrant et al., 2007; Wang et al., 2010; Song et al., 2011). Interestingly, SA and *Pseudomonas* infection cause DNA damage, such as DNA double strand breaks, suggesting that DNA damage response is an intrinsic component of SA-mediated transcription during plant immunity (Yan et al., 2013; Song and Bent, 2014).

Besides functional regulation of transcription factors by NPR1 through complex formation, NPR1 also controls expression of transcription factors, such as WRKY transcription factors, which are required for SA-mediated transcriptional reprogramming (Wang et al., 2006; Pajerowska-Mukhtar et al., 2012). The *Arabidopsis* genome encodes 74 WRKY factors which bind the specific DNA sequence (C/TTGACT/C), termed the W-box (Rushton et al., 2010). WRKY factors form a complex interconnected regulatory network, containing recurring regulatory patterns, such as both positive and negative feedback and feedforward loops. This WRKY network ensures rapid and efficient signal amplification and allows tight control to limit the plant immune response. Furthermore, the presence of multiple W-boxes in the *NPR1* promoter suggests regulation of *NPR1* expression by WRKY factors, which is indeed supported by NPR1 promoter analysis (Yu et al., 2001). Thus, WRKY transcriptional regulatory networks downstream of NPR1 amplify and fine-tune SA-mediated transcriptional reprogramming.

## COMPENSATION OF SA SIGNALING

The importance of SA signaling during immunity is reflected by the fact that pathogen effectors target it for virulence, either by preventing SA accumulation (Djamei et al., 2011; Rabe et al., 2013; Liu et al., 2014) or by dampen SA signaling and transcriptional regulation, using the antagonistic interaction between SA and JA signaling (Uppalapati et al., 2007; Caillaud et al., 2013; Jiang et al., 2013; Gimenez-Ibanez et al., 2014). It is reasonable to assume that plants have evolved compensatory mechanism(s) to circumvent weakened SA signaling upon effector attack, thereby ensuring robust immune response (Tsuda and Katagiri, 2010). For example, although it is believed that SA and JA signaling antagonize each other, a recent study suggests the compensation of SA accumulation by JA (Kim et al., 2014). The MAMP flg22 induces SA accumulation in an *ICS1*-dependent manner (Tsuda et al., 2008b). Additionally, a component of the SA amplification loop, PAD4, is required for full induction of SA (Zhou et al., 1998; Tsuda et al., 2008b). In agreement with the antagonistic relationship between JA and SA, single mutation in the JA biosynthesis gene *DDE2* leads to higher SA accumulation upon flg22 treatment. However, combined mutation in *DDE2* and *PAD4* diminishes SA accumulation comparable to that in *sid2*, suggesting that although JA suppresses SA accumulation through PAD4, it also supports SA accumulation once *PAD4* is compromised. Thus, JA signaling represents a compensation mechanism for SA accumulation during PTI.

In addition to JA, MAPK signaling also compensates SA signaling to secure transcriptional regulation of SA-responsive genes in ETI (Tsuda et al., 2013). Activation of the *Arabidopsis* MAPKs MPK3 and MPK6 is transient during PTI, but sustained during RPS2- and RPM1-mediated ETI (Tsuda et al., 2013), or upon *B. cinerea* infection (Han et al., 2010). While transient activation of MPK3 and MPK6 is not sufficient to overcome SA-dependency of a subset of SA-responsive genes such as *PR1*, prolonged activation of MPK3 and MPK6 facilitates their transcriptional regulation independent of SA. Furthermore, this compensation mechanism does not require NPR1 since *NPR1* mutation does not affect *PR1* induction mediated by prolonged MAPK activation. It can be assumed that prolonged MAPK activation bypasses the requirement of NPR1 to regulate transcription factor(s) involved in SA response. Although transcription factors shared by SA and the MAPK cascade are not known, large-scale protein target identifications of MPK3 and MPK6 would help to identify candidates (Popescu et al., 2009; Hoehenwarter et al., 2013). Among them, TGA transcription factors are reasonable candidates (Wang and Fobert, 2013). However, how this quantitative MAPK activation leads to qualitatively different transcriptional outputs still remains to be determined. One possible answer lays in temporal regulation of transcription factor(s). Hereby, the MAPKs first activate expression of transcription factor(s), and later on phosphorylate the accumulated transcription factor(s), representing a feedforward loop for activation of the transcription factor(s). In this case, only prolonged MAPK activation ensures activation of the transcription factor(s). Indeed, the MAPKs regulate expression of a diverse transcription factor set (Mao et al., 2011; Li et al., 2012; Meng et al., 2013; Tsuda et al., 2013; Frei dit Frey et al., 2014), but whether the MAPKs also phosphorylate them is a future issue.

## CONCLUSIONS AND PERSPECTIVES

Over the past decade a number of researches have shed light into our understanding of SA-mediated signaling, through the discoveries of calcium signaling as the major switch for SA biosynthesis, NPR family members as SA receptors, and the mechanism for NPR1-mediated transcriptional reprogramming. However, many questions are still unanswered, starting with identification of plant *IPL* gene(s) to further validate the IC pathway as the major route for SA biosynthesis in plants. The controversy for SA perception should also be solved in the future. In addition, information for temporal and spatial dynamics of SA biosynthesis and SA-mediated transcriptional reprogramming is missing. For this, systems approaches using time-series genomics data sets and tissue-specific analysis will help our conception (Mine et al., 2014). Most studies are based on experiments using the model plant *Arabidopsis*. Analysis of different plant species is necessary to understand evolutionary conservation and diversification of SA signal transduction. Finally, identification of the molecular components in MAPK-mediated SA/NPR1-independent gene regulation of SA-responsive genes in ETI will shed light on the molecular mechanism of SA compensation.

### Conflict of Interest Statement

The authors declare that the research was conducted in the absence of any commercial or financial relationships that could be construed as a potential conflict of interest.
